# Supplementary figures and images for: Genome-wide in silico identification of membrane-bound transcription factors in plant species
Source: PeerJ. 2017 Nov 15;5:e4051. doi: 10.7717/peerj.4051 (PMC5694209; doi:10.7717/peerj.4051)

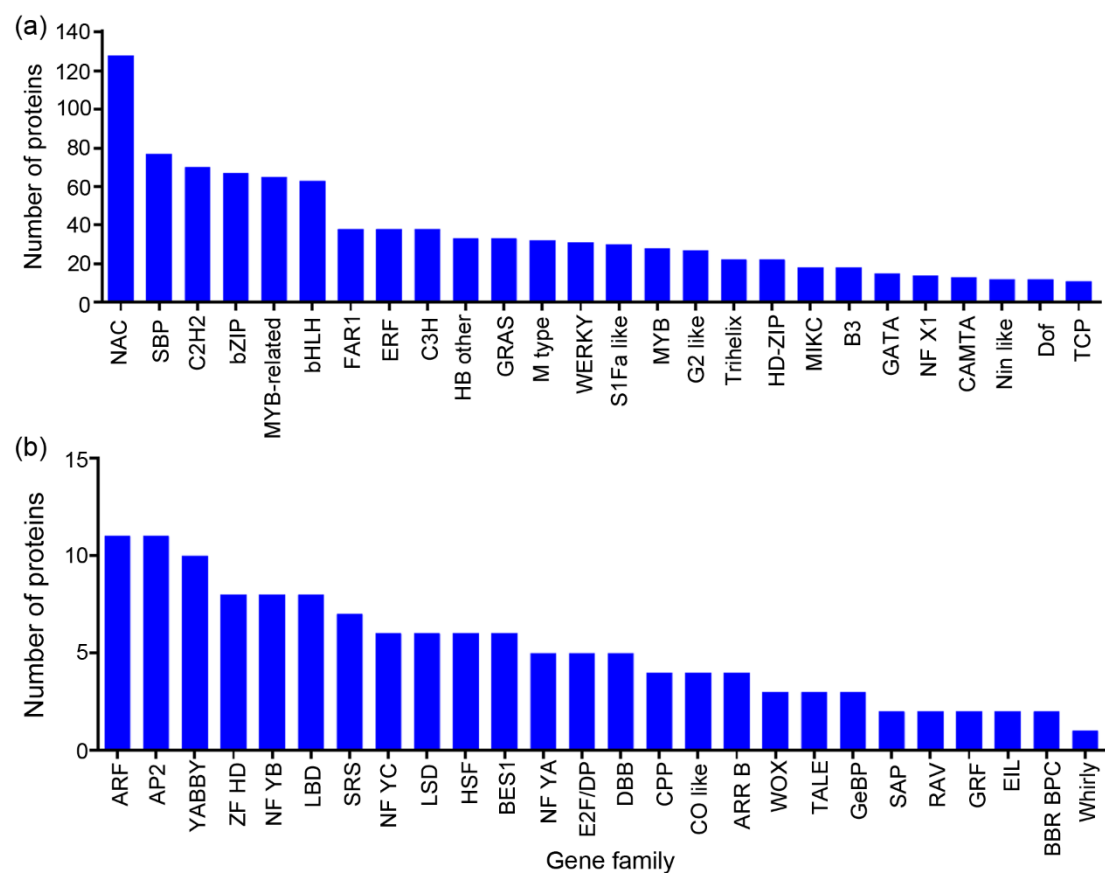

**Fig. S1.** Gene family of membrane-bound transcription factors in the 14 plant species.

Supplement: Figure S1 [file peerj-05-4051-s001.pdf]
