# Supplementary material for: Genome-wide in silico identification of membrane-bound transcription factors in plant species
Source: PeerJ. 2017 Nov 15;5:e4051. doi: 10.7717/peerj.4051 (PMC5694209; doi:10.7717/peerj.4051)
Supplement: Table S1 [file peerj-05-4051-s002.docx]

| TF family | Species | Gene | Function | Reference |
| --- | --- | --- | --- | --- |
| bZIP | *Arabidopsis* | AT1G42990 | ER stress | (Iwata and Koizumi, 2005; Iwata et al., 2008) |
| bZIP | *Arabidopsis* | AT2G40950 | Salt stress, BR signaling | (Liu et al., 2007a; Che et al., 2010) |
| bZIP | *Arabidopsis* | AT3G10800 | ER stress, heat stress, BR signaling | (Liu et al., 2007b; Gao et al., 2008; Che et al., 2010) |
| MYB related | *Arabidopsis* | AT5G45420 | Root hair development | (Slabaugh et al., 2011) |
| NAC | *Arabidopsis* | AT1G32870 | UV-B response; ANAC013, mitochondria retrograde signaling | (Safrany et al., 2008; De Clercq et al., 2013) |
| NAC | *Arabidopsis* | AT1G34190 | Mitochondria retrograde signaling | (Ng et al., 2013) |
| NAC | *Arabidopsis* | AT2G27300 | Salt responsive flowering, seed germination | (Kim et al., 2007; Kim et al., 2008) |
| NAC | *Arabidopsis* | AT3G10500 | ROS homeostasis | (Lee et al., 2012) |
| NAC | *Arabidopsis* | AT3G49530 | Cold stress, drought stress, ER stress | (Seo et al., 2010; Seo et al., 2010; Kim et al., 2012; Yang et al., 2014) |
| NAC | *Arabidopsis* | At4g01540 | Cell division | (Kim et al., 2006) |
| NAC | *Arabidopsis* | AT4G01550 | Seed germination | (Park et al., 2011) |
| NAC | *Arabidopsis* | AT4G35580 | Osmotic stress signaling | (Yoon et al., 2008) |
| NAC | *Arabidopsis* | AT5G22290 | Sugar signaling, ROS signaling, floral initiation, ER stress | (Li et al., 2010; Li et al., 2011; Klein et al., 2012; Yang et al., 2014) |

**Table S1**. Known functions of trans-membrane transcription factors in plant species.

**References:**

**Che, P., Bussell, J.D., Zhou, W., Estavillo, G.M., Pogson, B.J., and Smith, S.M.** (2010). Signaling from the endoplasmic reticulum activates brassinosteroid signaling and promotes acclimation to stress in Arabidopsis. Sci Signal **3,** a69.

**De Clercq, I., Vermeirssen, V., Van Aken, O., Vandepoele, K., Murcha, M.W., Law, S.R., Inze, A., Ng, S., Ivanova, A., Rombaut, D., van de Cotte, B., Jaspers, P., Van de Peer, Y., Kangasjarvi, J., Whelan, J., and Van Breusegem, F.** (2013). The membrane-bound NAC transcription factor ANAC013 functions in mitochondrial retrograde regulation of the oxidative stress response in Arabidopsis. Plant Cell **25,** 3472-3490.

**Gao, H., Brandizzi, F., Benning, C., and Larkin, R.M.** (2008). A membrane-tethered transcription factor defines a branch of the heat stress response in Arabidopsis thaliana. Proc Natl Acad Sci U S A **105,** 16398-16403.

**Iwata, Y., and Koizumi, N.** (2005). An Arabidopsis transcription factor, AtbZIP60, regulates the endoplasmic reticulum stress response in a manner unique to plants. Proc Natl Acad Sci U S A **102,** 5280-5285.

**Iwata, Y., Fedoroff, N.V., and Koizumi, N.** (2008). Arabidopsis bZIP60 is a proteolysis-activated transcription factor involved in the endoplasmic reticulum stress response. Plant Cell **20,** 3107-3121.

**Kim, M.J., Park, M.J., Seo, P.J., Song, J.S., Kim, H.J., and Park, C.M.** (2012). Controlled nuclear import of the transcription factor NTL6 reveals a cytoplasmic role of SnRK2.8 in the drought-stress response. Biochem J **448,** 353-363.

**Kim, S.G., Kim, S.Y., and Park, C.M.** (2007). A membrane-associated NAC transcription factor regulates salt-responsive flowering via FLOWERING LOCUS T in Arabidopsis. Planta **226,** 647-654.

**Kim, S.G., Lee, A.K., Yoon, H.K., and Park, C.M.** (2008). A membrane-bound NAC transcription factor NTL8 regulates gibberellic acid-mediated salt signaling in Arabidopsis seed germination. Plant J **55,** 77-88.

**Kim, Y.S., Kim, S.G., Park, J.E., Park, H.Y., Lim, M.H., Chua, N.H., and Park, C.M.** (2006). A membrane-bound NAC transcription factor regulates cell division in Arabidopsis. Plant Cell **18,** 3132-3144.

**Klein, P., Seidel, T., Stocker, B., and Dietz, K.J.** (2012). The membrane-tethered transcription factor ANAC089 serves as redox-dependent suppressor of stromal ascorbate peroxidase gene expression. Front Plant Sci **3,** 247.

**Lee, S., Seo, P.J., Lee, H.J., and Park, C.M.** (2012). A NAC transcription factor NTL4 promotes reactive oxygen species production during drought-induced leaf senescence in Arabidopsis. Plant J **70,** 831-844.

**Li, J., Zhang, J., Wang, X., and Chen, J.** (2010). A membrane-tethered transcription factor ANAC089 negatively regulates floral initiation in Arabidopsis thaliana. Sci China Life Sci **53,** 1299-1306.

**Li, P., Wind, J.J., Shi, X., Zhang, H., Hanson, J., Smeekens, S.C., and Teng, S.** (2011). Fructose sensitivity is suppressed in Arabidopsis by the transcription factor ANAC089 lacking the membrane-bound domain. Proc Natl Acad Sci U S A **108,** 3436-3441.

**Liu, J.X., Srivastava, R., Che, P., and Howell, S.H.** (2007a). Salt stress responses in Arabidopsis utilize a signal transduction pathway related to endoplasmic reticulum stress signaling. Plant J **51,** 897-909.

**Liu, J.X., Srivastava, R., Che, P., and Howell, S.H.** (2007b). An endoplasmic reticulum stress response in Arabidopsis is mediated by proteolytic processing and nuclear relocation of a membrane-associated transcription factor, bZIP28. Plant Cell **19,** 4111-4119.

**Ng, S., Ivanova, A., Duncan, O., Law, S.R., Van Aken, O., De Clercq, I., Wang, Y., Carrie, C., Xu, L., Kmiec, B., Walker, H., Van Breusegem, F., Whelan, J., and Giraud, E.** (2013). A membrane-bound NAC transcription factor, ANAC017, mediates mitochondrial retrograde signaling in Arabidopsis. Plant Cell **25,** 3450-3471.

**Park, J., Kim, Y.S., Kim, S.G., Jung, J.H., Woo, J.C., and Park, C.M.** (2011). Integration of auxin and salt signals by the NAC transcription factor NTM2 during seed germination in Arabidopsis. Plant Physiol **156,** 537-549.

**Safrany, J., Haasz, V., Mate, Z., Ciolfi, A., Feher, B., Oravecz, A., Stec, A., Dallmann, G., Morelli, G., Ulm, R., and Nagy, F.** (2008). Identification of a novel cis-regulatory element for UV-B-induced transcription in Arabidopsis. Plant J **54,** 402-414.

**Seo, P.J., Kim, M.J., Song, J.S., Kim, Y.S., Kim, H.J., and Park, C.M.** (2010). Proteolytic processing of an Arabidopsis membrane-bound NAC transcription factor is triggered by cold-induced changes in membrane fluidity. Biochem J **427,** 359-367.

**Seo, P.J., Kim, M.J., Park, J.Y., Kim, S.Y., Jeon, J., Lee, Y.H., Kim, J., and Park, C.M.** (2010). Cold activation of a plasma membrane-tethered NAC transcription factor induces a pathogen resistance response in Arabidopsis. Plant J **61,** 661-671.

**Slabaugh, E., Held, M., and Brandizzi, F.** (2011). Control of root hair development in Arabidopsis thaliana by an endoplasmic reticulum anchored member of the R2R3-MYB transcription factor family. Plant J **67,** 395-405.

**Yang, Z.T., Lu, S.J., Wang, M.J., Bi, D.L., Sun, L., Zhou, S.F., Song, Z.T., and Liu, J.X.** (2014). A plasma membrane-tethered transcription factor, NAC062/ANAC062/NTL6, mediates the unfolded protein response in Arabidopsis. Plant J.

**Yang, Z.T., Wang, M.J., Sun, L., Lu, S.J., Bi, D.L., Sun, L., Song, Z.T., Zhang, S.S., Zhou, S.F., and Liu, J.X.** (2014). The membrane-associated transcription factor NAC089 controls ER-stress-induced programmed cell death in plants. PLoS Genet **10,** e1004243.

**Yoon, H.K., Kim, S.G., Kim, S.Y., and Park, C.M.** (2008). Regulation of leaf senescence by NTL9-mediated osmotic stress signaling in Arabidopsis. Mol Cells **25,** 438-445.
