# Supplementary material for: Genome-wide in silico identification of membrane-bound transcription factors in plant species
Source: PeerJ. 2017 Nov 15;5:e4051. doi: 10.7717/peerj.4051 (PMC5694209; doi:10.7717/peerj.4051)
Supplement: Table S3 [file peerj-05-4051-s004.docx]

**Table S3**. Transcripts of membrane-bound transcription factors in *Arabidopsis thaliana* and *Gossypium raimondii*.

| **Species** | **Loci** | **Transcript** | **Gene family** | **TM domain** |
| --- | --- | --- | --- | --- |
| *Arabidopsis thaliana* | AT1G01010 | AT1G01010.1 | NAC | YES |
| *Arabidopsis thaliana* | AT1G08465 | AT1G08465.1 | YABBY | YES |
| *Arabidopsis thaliana* | AT1G20980 | AT1G20980.1 | SBP | YES |
| *Arabidopsis thaliana* | AT1G23420 | AT1G23420.1 | YABBY | YES |
| *Arabidopsis thaliana* | AT1G28450 | AT1G28450.1 | M-type | YES |
| *Arabidopsis thaliana* | AT1G32540 | AT1G32540.1 | LSD | YES |
| *Arabidopsis thaliana* | AT1G32540 | AT1G32540.2 | LSD | NO |
| *Arabidopsis thaliana* | AT1G32540 | AT1G32540.3 | LSD | NO |
| *Arabidopsis thaliana* | AT1G32870 | AT1G32870.1 | NAC | YES |
| *Arabidopsis thaliana* | AT1G32870 | AT1G32870.2 | NAC | YES |
| *Arabidopsis thaliana* | AT1G33060 | AT1G33060.1 | NAC | YES |
| *Arabidopsis thaliana* | AT1G33060 | AT1G33060.2 | NAC | YES |
| *Arabidopsis thaliana* | AT1G34180 | AT1G34180.1 | NAC | YES |
| *Arabidopsis thaliana* | AT1G34180 | AT1G34180.2 | NAC | YES |
| *Arabidopsis thaliana* | AT1G34190 | AT1G34190.1 | NAC | YES |
| *Arabidopsis thaliana* | AT1G42990 | AT1G42990.1 | bZIP | YES |
| *Arabidopsis thaliana* | AT1G51070 | AT1G51070.1 | bHLH | NO |
| *Arabidopsis thaliana* | AT1G51070 | AT1G51070.2 | bHLH | YES |
| *Arabidopsis thaliana* | AT1G65910 | AT1G65910.1 | NAC | YES |
| *Arabidopsis thaliana* | AT1G76580 | AT1G76580.1 | SBP | YES |
| *Arabidopsis thaliana* | AT2G13960 | AT2G13960.1 | MYB_related | NO |
| *Arabidopsis thaliana* | AT2G13960 | AT2G13960.2 | MYB_related | YES |
| *Arabidopsis thaliana* | AT2G27300 | AT2G27300.1 | NAC | YES |
| *Arabidopsis thaliana* | AT2G29065 | AT2G29065.1 | GRAS | YES |
| *Arabidopsis thaliana* | AT2G29660 | AT2G29660.1 | C2H2 | YES |
| *Arabidopsis thaliana* | AT2G31280 | AT2G31280.1 | bHLH | NO |
| *Arabidopsis thaliana* | AT2G31280 | AT2G31280.2 | bHLH | YES |
| *Arabidopsis thaliana* | AT2G31280 | AT2G31280.3 | bHLH | NO |
| *Arabidopsis thaliana* | AT2G32930 | AT2G32930.1 | C3H | NO |
| *Arabidopsis thaliana* | AT2G32930 | AT2G32930.2 | C3H | YES |
| *Arabidopsis thaliana* | AT2G37120 | AT2G37120.1 | S1Fa-like | YES |
| *Arabidopsis thaliana* | AT2G40950 | AT2G40950.1 | bZIP | YES |
| *Arabidopsis thaliana* | AT2G47070 | AT2G47070.1 | SBP | YES |
| *Arabidopsis thaliana* | AT3G01140 | AT3G01140.1 | MYB | YES |
| *Arabidopsis thaliana* | AT3G04930 | AT3G04930.1 | GeBP | NO |
| *Arabidopsis thaliana* | AT3G04930 | AT3G04930.2 | GeBP | YES |
| *Arabidopsis thaliana* | AT3G09735 | AT3G09735.1 | S1Fa-like | YES |
| *Arabidopsis thaliana* | AT3G10480 | AT3G10480.1 | NAC | NO |
| *Arabidopsis thaliana* | AT3G10480 | AT3G10480.2 | NAC | NO |
| *Arabidopsis thaliana* | AT3G10480 | AT3G10480.3 | NAC | YES |
| *Arabidopsis thaliana* | AT3G10500 | AT3G10500.1 | NAC | YES |
| *Arabidopsis thaliana* | AT3G10800 | AT3G10800.1 | bZIP | YES |
| *Arabidopsis thaliana* | AT3G11580 | AT3G11580.1 | B3 | NO |
| *Arabidopsis thaliana* | AT3G11580 | AT3G11580.2 | B3 | YES |
| *Arabidopsis thaliana* | AT3G20880 | AT3G20880.1 | C2H2 | YES |
| *Arabidopsis thaliana* | AT3G44290 | AT3G44290.1 | NAC | YES |
| *Arabidopsis thaliana* | AT3G47870 | AT3G47870.1 | LBD | YES |
| *Arabidopsis thaliana* | AT3G49530 | AT3G49530.1 | NAC | YES |
| *Arabidopsis thaliana* | AT3G53370 | AT3G53370.1 | S1Fa-like | YES |
| *Arabidopsis thaliana* | AT3G53370 | AT3G53370.2 | S1Fa-like | NO |
| *Arabidopsis thaliana* | AT3G55370 | AT3G55370.1 | Dof | NO |
| *Arabidopsis thaliana* | AT3G55370 | AT3G55370.2 | Dof | NO |
| *Arabidopsis thaliana* | AT3G55370 | AT3G55370.3 | Dof | YES |
| *Arabidopsis thaliana* | AT3G56660 | AT3G56660.1 | bZIP | YES |
| *Arabidopsis thaliana* | AT3G60030 | AT3G60030.1 | SBP | YES |
| *Arabidopsis thaliana* | AT4G01540 | AT4G01540.1 | NAC | YES |
| *Arabidopsis thaliana* | AT4G01540 | AT4G01540.2 | NAC | YES |
| *Arabidopsis thaliana* | AT4G01550 | AT4G01550.1 | NAC | YES |
| *Arabidopsis thaliana* | AT4G02560 | AT4G02560.1 | HB-other | YES |
| *Arabidopsis thaliana* | AT4G02560 | AT4G02560.2 | HB-other | YES |
| *Arabidopsis thaliana* | AT4G12850 | AT4G12850.1 | FAR1 | NO |
| *Arabidopsis thaliana* | AT4G12850 | AT4G12850.2 | FAR1 | YES |
| *Arabidopsis thaliana* | AT4G16150 | AT4G16150.1 | CAMTA | YES |
| *Arabidopsis thaliana* | AT4G35580 | AT4G35580.1 | NAC | YES |
| *Arabidopsis thaliana* | AT4G35580 | AT4G35580.2 | NAC | NO |
| *Arabidopsis thaliana* | AT4G35580 | AT4G35580.3 | NAC | NO |
| *Arabidopsis thaliana* | AT4G37180 | AT4G37180.1 | G2-like | NO |
| *Arabidopsis thaliana* | AT4G37180 | AT4G37180.2 | G2-like | YES |
| *Arabidopsis thaliana* | AT4G37730 | AT4G37730.1 | bZIP | YES |
| *Arabidopsis thaliana* | AT4G38070 | AT4G38070.1 | bHLH | YES |
| *Arabidopsis thaliana* | AT4G38340 | AT4G38340.1 | Nin-like | YES |
| *Arabidopsis thaliana* | AT5G04400 | AT5G04400.1 | NAC | YES |
| *Arabidopsis thaliana* | AT5G04410 | AT5G04410.1 | NAC | YES |
| *Arabidopsis thaliana* | AT5G05660 | AT5G05660.1 | NF-X1 | YES |
| *Arabidopsis thaliana* | AT5G09410 | AT5G09410.1 | CAMTA | NO |
| *Arabidopsis thaliana* | AT5G09410 | AT5G09410.2 | CAMTA | NO |
| *Arabidopsis thaliana* | AT5G09410 | AT5G09410.3 | CAMTA | YES |
| *Arabidopsis thaliana* | AT5G10510 | AT5G10510.3 | AP2 | YES |
| *Arabidopsis thaliana* | AT5G14280 | AT5G14280.1 | GeBP | YES |
| *Arabidopsis thaliana* | AT5G18830 | AT5G18830.1 | SBP | YES |
| *Arabidopsis thaliana* | AT5G18830 | AT5G18830.2 | SBP | YES |
| *Arabidopsis thaliana* | AT5G18830 | AT5G18830.3 | SBP | YES |
| *Arabidopsis thaliana* | AT5G22290 | AT5G22290.1 | NAC | YES |
| *Arabidopsis thaliana* | AT5G25475 | AT5G25475.1 | B3 | NO |
| *Arabidopsis thaliana* | AT5G25475 | AT5G25475.2 | B3 | NO |
| *Arabidopsis thaliana* | AT5G25475 | AT5G25475.3 | B3 | NO |
| *Arabidopsis thaliana* | AT5G25475 | AT5G25475.4 | B3 | YES |
| *Arabidopsis thaliana* | AT5G26580 | AT5G26580.1 | M-type | YES |
| *Arabidopsis thaliana* | AT5G33210 | AT5G33210.1 | SRS | YES |
| *Arabidopsis thaliana* | AT5G33210 | AT5G33210.2 | SRS | NO |
| *Arabidopsis thaliana* | AT5G44180 | AT5G44180.1 | HB-other | NO |
| *Arabidopsis thaliana* | AT5G44180 | AT5G44180.2 | HB-other | YES |
| *Arabidopsis thaliana* | AT5G45420 | AT5G45420.1 | MYB_related | YES |
| *Arabidopsis thaliana* | AT5G57150 | AT5G57150.1 | bHLH | NO |
| *Arabidopsis thaliana* | AT5G57150 | AT5G57150.2 | bHLH | YES |
| *Arabidopsis thaliana* | AT5G57150 | AT5G57150.3 | bHLH | NO |
| *Arabidopsis thaliana* | AT5G57150 | AT5G57150.4 | bHLH | YES |
| *Arabidopsis thaliana* | AT5G63280 | AT5G63280.1 | C2H2 | YES |
| *Arabidopsis thaliana* | AT5G65070 | AT5G65070.1 | MIKC | YES |
| *Arabidopsis thaliana* | AT5G65070 | AT5G65070.2 | MIKC | YES |
| *Arabidopsis thaliana* | AT5G65070 | AT5G65070.3 | MIKC | YES |
| *Gossypium raimondii* | Gorai.001G003200 | Gorai.001G003200.1 | S1Fa-like | YES |
| *Gossypium raimondii* | Gorai.001G047400 | Gorai.001G047400.1 | Trihelix | YES |
| *Gossypium raimondii* | Gorai.001G061600 | Gorai.001G061600.1 | C2H2 | YES |
| *Gossypium raimondii* | Gorai.001G061900 | Gorai.001G061900.1 | C2H2 | YES |
| *Gossypium raimondii* | Gorai.001G064700 | Gorai.001G064700.1 | NF-YB | NO |
| *Gossypium raimondii* | Gorai.001G064700 | Gorai.001G064700.2 | NF-YB | NO |
| *Gossypium raimondii* | Gorai.001G064700 | Gorai.001G064700.3 | NF-YB | NO |
| *Gossypium raimondii* | Gorai.001G064700 | Gorai.001G064700.4 | NF-YB | NO |
| *Gossypium raimondii* | Gorai.001G064700 | Gorai.001G064700.5 | NF-YB | YES |
| *Gossypium raimondii* | Gorai.001G073600 | Gorai.001G073600.1 | HD-ZIP | YES |
| *Gossypium raimondii* | Gorai.001G073600 | Gorai.001G073600.2 | HD-ZIP | NO |
| *Gossypium raimondii* | Gorai.001G073600 | Gorai.001G073600.3 | HD-ZIP | NO |
| *Gossypium raimondii* | Gorai.001G073600 | Gorai.001G073600.4 | HD-ZIP | NO |
| *Gossypium raimondii* | Gorai.001G115200 | Gorai.001G115200.1 | C2H2 | YES |
| *Gossypium raimondii* | Gorai.001G117100 | Gorai.001G117100.1 | C2H2 | YES |
| *Gossypium raimondii* | Gorai.001G129500 | Gorai.001G129500.1 | Dof | YES |
| *Gossypium raimondii* | Gorai.001G150000 | Gorai.001G150000.1 | NAC | YES |
| *Gossypium raimondii* | Gorai.001G150000 | Gorai.001G150000.2 | NAC | YES |
| *Gossypium raimondii* | Gorai.001G150000 | Gorai.001G150000.3 | NAC | NO |
| *Gossypium raimondii* | Gorai.001G150000 | Gorai.001G150000.4 | NAC | YES |
| *Gossypium raimondii* | Gorai.001G150000 | Gorai.001G150000.5 | NAC | NO |
| *Gossypium raimondii* | Gorai.001G150000 | Gorai.001G150000.6 | NAC | NO |
| *Gossypium raimondii* | Gorai.001G153900 | Gorai.001G153900.1 | ARF | NO |
| *Gossypium raimondii* | Gorai.001G153900 | Gorai.001G153900.2 | ARF | NO |
| *Gossypium raimondii* | Gorai.001G153900 | Gorai.001G153900.3 | ARF | NO |
| *Gossypium raimondii* | Gorai.001G153900 | Gorai.001G153900.4 | ARF | NO |
| *Gossypium raimondii* | Gorai.001G153900 | Gorai.001G153900.5 | ARF | NO |
| *Gossypium raimondii* | Gorai.001G153900 | Gorai.001G153900.6 | ARF | NO |
| *Gossypium raimondii* | Gorai.001G153900 | Gorai.001G153900.7 | ARF | YES |
| *Gossypium raimondii* | Gorai.001G171000 | Gorai.001G171000.1 | HD-ZIP | NO |
| *Gossypium raimondii* | Gorai.001G171000 | Gorai.001G171000.2 | HD-ZIP | NO |
| *Gossypium raimondii* | Gorai.001G171000 | Gorai.001G171000.3 | HD-ZIP | YES |
| *Gossypium raimondii* | Gorai.001G171000 | Gorai.001G171000.4 | HD-ZIP | NO |
| *Gossypium raimondii* | Gorai.001G171000 | Gorai.001G171000.5 | HD-ZIP | NO |
| *Gossypium raimondii* | Gorai.001G171000 | Gorai.001G171000.6 | HD-ZIP | NO |
| *Gossypium raimondii* | Gorai.001G191800 | Gorai.001G191800.1 | SAP | YES |
| *Gossypium raimondii* | Gorai.002G112200 | Gorai.002G112200.1 | SBP | YES |
| *Gossypium raimondii* | Gorai.002G123600 | Gorai.002G123600.1 | C2H2 | YES |
| *Gossypium raimondii* | Gorai.002G242000 | Gorai.002G242000.1 | Trihelix | YES |
| *Gossypium raimondii* | Gorai.002G242000 | Gorai.002G242000.2 | Trihelix | NO |
| *Gossypium raimondii* | Gorai.002G247000 | Gorai.002G247000.1 | E2F/DP | NO |
| *Gossypium raimondii* | Gorai.002G247000 | Gorai.002G247000.2 | E2F/DP | NO |
| *Gossypium raimondii* | Gorai.002G247000 | Gorai.002G247000.3 | E2F/DP | NO |
| *Gossypium raimondii* | Gorai.002G247000 | Gorai.002G247000.4 | E2F/DP | NO |
| *Gossypium raimondii* | Gorai.002G247000 | Gorai.002G247000.5 | E2F/DP | YES |
| *Gossypium raimondii* | Gorai.002G247000 | Gorai.002G247000.6 | E2F/DP | NO |
| *Gossypium raimondii* | Gorai.002G247800 | Gorai.002G247800.1 | MYB_related | NO |
| *Gossypium raimondii* | Gorai.002G247800 | Gorai.002G247800.2 | MYB_related | NO |
| *Gossypium raimondii* | Gorai.002G247800 | Gorai.002G247800.3 | MYB_related | YES |
| *Gossypium raimondii* | Gorai.002G247800 | Gorai.002G247800.4 | MYB_related | NO |
| *Gossypium raimondii* | Gorai.002G249300 | Gorai.002G249300.1 | S1Fa-like | YES |
| *Gossypium raimondii* | Gorai.002G249300 | Gorai.002G249300.2 | S1Fa-like | YES |
| *Gossypium raimondii* | Gorai.003G073300 | Gorai.003G073300.1 | NAC | YES |
| *Gossypium raimondii* | Gorai.003G073300 | Gorai.003G073300.2 | NAC | NO |
| *Gossypium raimondii* | Gorai.003G180100 | Gorai.003G180100.1 | ARR-B | YES |
| *Gossypium raimondii* | Gorai.003G180100 | Gorai.003G180100.2 | ARR-B | NO |
| *Gossypium raimondii* | Gorai.003G180100 | Gorai.003G180100.3 | ARR-B | NO |
| *Gossypium raimondii* | Gorai.004G014800 | Gorai.004G014800.1 | SRS | YES |
| *Gossypium raimondii* | Gorai.004G034900 | Gorai.004G034900.1 | bHLH | NO |
| *Gossypium raimondii* | Gorai.004G035300 | Gorai.004G035300.1 | bHLH | NO |
| *Gossypium raimondii* | Gorai.004G035800 | Gorai.004G035800.1 | bHLH | NO |
| *Gossypium raimondii* | Gorai.004G035800 | Gorai.004G035800.2 | bHLH | NO |
| *Gossypium raimondii* | Gorai.004G035800 | Gorai.004G035800.3 | bHLH | NO |
| *Gossypium raimondii* | Gorai.004G035800 | Gorai.004G035800.4 | bHLH | YES |
| *Gossypium raimondii* | Gorai.004G047200 | Gorai.004G047200.1 | SBP | YES |
| *Gossypium raimondii* | Gorai.004G047200 | Gorai.004G047200.2 | SBP | YES |
| *Gossypium raimondii* | Gorai.004G048200 | Gorai.004G048200.1 | NAC | YES |
| *Gossypium raimondii* | Gorai.004G120700 | Gorai.004G120700.1 | HD-ZIP | YES |
| *Gossypium raimondii* | Gorai.004G149000 | Gorai.004G149000.1 | NAC | NO |
| *Gossypium raimondii* | Gorai.004G149000 | Gorai.004G149000.2 | NAC | NO |
| *Gossypium raimondii* | Gorai.004G149000 | Gorai.004G149000.3 | NAC | NO |
| *Gossypium raimondii* | Gorai.004G149000 | Gorai.004G149000.4 | NAC | NO |
| *Gossypium raimondii* | Gorai.004G149000 | Gorai.004G149000.5 | NAC | NO |
| *Gossypium raimondii* | Gorai.004G149000 | Gorai.004G149000.6 | NAC | NO |
| *Gossypium raimondii* | Gorai.004G149000 | Gorai.004G149000.7 | NAC | NO |
| *Gossypium raimondii* | Gorai.004G149000 | Gorai.004G149000.8 | NAC | NO |
| *Gossypium raimondii* | Gorai.004G149000 | Gorai.004G149000.9 | NAC | YES |
| *Gossypium raimondii* | Gorai.004G218300 | Gorai.004G218300.1 | bZIP | NO |
| *Gossypium raimondii* | Gorai.004G218300 | Gorai.004G218300.2 | bZIP | YES |
| *Gossypium raimondii* | Gorai.004G218300 | Gorai.004G218300.3 | bZIP | NO |
| *Gossypium raimondii* | Gorai.004G218300 | Gorai.004G218300.4 | bZIP | NO |
| *Gossypium raimondii* | Gorai.004G218300 | Gorai.004G218300.5 | bZIP | NO |
| *Gossypium raimondii* | Gorai.004G218300 | Gorai.004G218300.6 | bZIP | NO |
| *Gossypium raimondii* | Gorai.005G016700 | Gorai.005G016700.1 | FAR1 | YES |
| *Gossypium raimondii* | Gorai.005G016700 | Gorai.005G016700.2 | FAR1 | NO |
| *Gossypium raimondii* | Gorai.005G016700 | Gorai.005G016700.3 | FAR1 | NO |
| *Gossypium raimondii* | Gorai.005G016700 | Gorai.005G016700.4 | FAR1 | NO |
| *Gossypium raimondii* | Gorai.005G056500 | Gorai.005G056500.1 | SBP | YES |
| *Gossypium raimondii* | Gorai.005G056500 | Gorai.005G056500.2 | SBP | NO |
| *Gossypium raimondii* | Gorai.005G056500 | Gorai.005G056500.3 | SBP | YES |
| *Gossypium raimondii* | Gorai.005G071400 | Gorai.005G071400.1 | SBP | YES |
| *Gossypium raimondii* | Gorai.005G071400 | Gorai.005G071400.2 | SBP | YES |
| *Gossypium raimondii* | Gorai.005G071400 | Gorai.005G071400.3 | SBP | YES |
| *Gossypium raimondii* | Gorai.005G076200 | Gorai.005G076200.1 | NAC | YES |
| *Gossypium raimondii* | Gorai.005G076200 | Gorai.005G076200.2 | NAC | YES |
| *Gossypium raimondii* | Gorai.005G076200 | Gorai.005G076200.4 | NAC | YES |
| *Gossypium raimondii* | Gorai.005G076200 | Gorai.005G076200.5 | NAC | YES |
| *Gossypium raimondii* | Gorai.005G076200 | Gorai.005G076200.6 | NAC | YES |
| *Gossypium raimondii* | Gorai.005G087600 | Gorai.005G087600.1 | MIKC | YES |
| *Gossypium raimondii* | Gorai.005G087600 | Gorai.005G087600.2 | MIKC | YES |
| *Gossypium raimondii* | Gorai.005G133400 | Gorai.005G133400.1 | Nin-like | YES |
| *Gossypium raimondii* | Gorai.005G134200 | Gorai.005G134200.1 | FAR1 | YES |
| *Gossypium raimondii* | Gorai.005G134200 | Gorai.005G134200.2 | FAR1 | NO |
| *Gossypium raimondii* | Gorai.005G134200 | Gorai.005G134200.3 | FAR1 | NO |
| *Gossypium raimondii* | Gorai.005G134200 | Gorai.005G134200.4 | FAR1 | NO |
| *Gossypium raimondii* | Gorai.005G134200 | Gorai.005G134200.5 | FAR1 | NO |
| *Gossypium raimondii* | Gorai.005G134200 | Gorai.005G134200.6 | FAR1 | NO |
| *Gossypium raimondii* | Gorai.005G134200 | Gorai.005G134200.7 | FAR1 | NO |
| *Gossypium raimondii* | Gorai.005G134200 | Gorai.005G134200.8 | FAR1 | NO |
| *Gossypium raimondii* | Gorai.005G134200 | Gorai.005G134200.9 | FAR1 | NO |
| *Gossypium raimondii* | Gorai.005G139600 | Gorai.005G139600.1 | HD-ZIP | NO |
| *Gossypium raimondii* | Gorai.005G139600 | Gorai.005G139600.2 | HD-ZIP | NO |
| *Gossypium raimondii* | Gorai.005G139600 | Gorai.005G139600.3 | HD-ZIP | NO |
| *Gossypium raimondii* | Gorai.005G139600 | Gorai.005G139600.4 | HD-ZIP | YES |
| *Gossypium raimondii* | Gorai.005G158800 | Gorai.005G158800.1 | C2H2 | NO |
| *Gossypium raimondii* | Gorai.005G158800 | Gorai.005G158800.2 | C2H2 | NO |
| *Gossypium raimondii* | Gorai.005G158800 | Gorai.005G158800.3 | C2H2 | NO |
| *Gossypium raimondii* | Gorai.005G158800 | Gorai.005G158800.4 | C2H2 | YES |
| *Gossypium raimondii* | Gorai.005G226700 | Gorai.005G226700.1 | C2H2 | YES |
| *Gossypium raimondii* | Gorai.005G226700 | Gorai.005G226700.2 | C2H2 | YES |
| *Gossypium raimondii* | Gorai.005G226700 | Gorai.005G226700.3 | C2H2 | YES |
| *Gossypium raimondii* | Gorai.005G226700 | Gorai.005G226700.4 | C2H2 | NO |
| *Gossypium raimondii* | Gorai.006G079000 | Gorai.006G079000.1 | CAMTA | YES |
| *Gossypium raimondii* | Gorai.006G087600 | Gorai.006G087600.1 | B3 | YES |
| *Gossypium raimondii* | Gorai.006G107500 | Gorai.006G107500.1 | ERF | YES |
| *Gossypium raimondii* | Gorai.006G113700 | Gorai.006G113700.1 | NAC | YES |
| *Gossypium raimondii* | Gorai.006G168100 | Gorai.006G168100.1 | S1Fa-like | YES |
| *Gossypium raimondii* | Gorai.006G192400 | Gorai.006G192400.1 | MYB |  |
| *Gossypium raimondii* | Gorai.006G203800 | Gorai.006G203800.1 | NAC | YES |
| *Gossypium raimondii* | Gorai.006G203800 | Gorai.006G203800.2 | NAC | NO |
| *Gossypium raimondii* | Gorai.006G203800 | Gorai.006G203800.3 | NAC | NO |
| *Gossypium raimondii* | Gorai.006G203800 | Gorai.006G203800.4 | NAC | NO |
| *Gossypium raimondii* | Gorai.006G203800 | Gorai.006G203800.5 | NAC | NO |
| *Gossypium raimondii* | Gorai.006G203800 | Gorai.006G203800.6 | NAC | NO |
| *Gossypium raimondii* | Gorai.006G270100 | Gorai.006G270100.1 | C2H2 | YES |
| *Gossypium raimondii* | Gorai.007G017500 | Gorai.007G017500.1 | NAC | YES |
| *Gossypium raimondii* | Gorai.007G017500 | Gorai.007G017500.2 | NAC | YES |
| *Gossypium raimondii* | Gorai.007G017500 | Gorai.007G017500.3 | NAC | YES |
| *Gossypium raimondii* | Gorai.007G099100 | Gorai.007G099100.1 | BES1 | NO |
| *Gossypium raimondii* | Gorai.007G099100 | Gorai.007G099100.2 | BES1 | NO |
| *Gossypium raimondii* | Gorai.007G099100 | Gorai.007G099100.3 | BES1 | NO |
| *Gossypium raimondii* | Gorai.007G099100 | Gorai.007G099100.4 | BES1 | NO |
| *Gossypium raimondii* | Gorai.007G099100 | Gorai.007G099100.5 | BES1 | NO |
| *Gossypium raimondii* | Gorai.007G099100 | Gorai.007G099100.6 | BES1 | NO |
| *Gossypium raimondii* | Gorai.007G099100 | Gorai.007G099100.7 | BES1 | YES |
| *Gossypium raimondii* | Gorai.007G099100 | Gorai.007G099100.8 | BES1 | YES |
| *Gossypium raimondii* | Gorai.007G109500 | Gorai.007G109500.1 | ARF | NO |
| *Gossypium raimondii* | Gorai.007G109500 | Gorai.007G109500.2 | ARF | NO |
| *Gossypium raimondii* | Gorai.007G109500 | Gorai.007G109500.3 | ARF | YES |
| *Gossypium raimondii* | Gorai.007G109500 | Gorai.007G109500.4 | ARF | NO |
| *Gossypium raimondii* | Gorai.007G109500 | Gorai.007G109500.5 | ARF | NO |
| *Gossypium raimondii* | Gorai.007G109500 | Gorai.007G109500.6 | ARF | NO |
| *Gossypium raimondii* | Gorai.007G109500 | Gorai.007G109500.7 | ARF | NO |
| *Gossypium raimondii* | Gorai.007G109500 | Gorai.007G109500.8 | ARF | NO |
| *Gossypium raimondii* | Gorai.007G109500 | Gorai.007G109500.9 | ARF | NO |
| *Gossypium raimondii* | Gorai.007G114500 | Gorai.007G114500.1 | NAC | YES |
| *Gossypium raimondii* | Gorai.007G114500 | Gorai.007G114500.2 | NAC | NO |
| *Gossypium raimondii* | Gorai.007G114500 | Gorai.007G114500.3 | NAC | NO |
| *Gossypium raimondii* | Gorai.007G114500 | Gorai.007G114500.4 | NAC | YES |
| *Gossypium raimondii* | Gorai.007G114500 | Gorai.007G114500.5 | NAC | YES |
| *Gossypium raimondii* | Gorai.007G114500 | Gorai.007G114500.6 | NAC | YES |
| *Gossypium raimondii* | Gorai.007G114500 | Gorai.007G114500.7 | NAC | YES |
| *Gossypium raimondii* | Gorai.007G114500 | Gorai.007G114500.8 | NAC | YES |
| *Gossypium raimondii* | Gorai.007G116100 | Gorai.007G116100.1 | SBP | YES |
| *Gossypium raimondii* | Gorai.007G188800 | Gorai.007G188800.1 | NAC | YES |
| *Gossypium raimondii* | Gorai.007G342200 | Gorai.007G342200.1 | MYB_related | NO |
| *Gossypium raimondii* | Gorai.007G342200 | Gorai.007G342200.2 | MYB_related | NO |
| *Gossypium raimondii* | Gorai.007G342200 | Gorai.007G342200.3 | MYB_related | NO |
| *Gossypium raimondii* | Gorai.007G342200 | Gorai.007G342200.4 | MYB_related | NO |
| *Gossypium raimondii* | Gorai.007G342200 | Gorai.007G342200.5 | MYB_related | NO |
| *Gossypium raimondii* | Gorai.007G342200 | Gorai.007G342200.6 | MYB_related | NO |
| *Gossypium raimondii* | Gorai.007G342200 | Gorai.007G342200.7 | MYB_related | YES |
| *Gossypium raimondii* | Gorai.008G060100 | Gorai.008G060100.1 | MYB | YES |
| *Gossypium raimondii* | Gorai.008G089900 | Gorai.008G089900.1 | CAMTA | NO |
| *Gossypium raimondii* | Gorai.008G089900 | Gorai.008G089900.2 | CAMTA | NO |
| *Gossypium raimondii* | Gorai.008G089900 | Gorai.008G089900.3 | CAMTA | NO |
| *Gossypium raimondii* | Gorai.008G089900 | Gorai.008G089900.4 | CAMTA | YES |
| *Gossypium raimondii* | Gorai.008G131800 | Gorai.008G131800.1 | MYB_related | YES |
| *Gossypium raimondii* | Gorai.008G131800 | Gorai.008G131800.2 | MYB_related | NO |
| *Gossypium raimondii* | Gorai.008G131800 | Gorai.008G131800.3 | MYB_related | YES |
| *Gossypium raimondii* | Gorai.008G131800 | Gorai.008G131800.4 | MYB_related | YES |
| *Gossypium raimondii* | Gorai.008G132000 | Gorai.008G132000.1 | MYB_related | YES |
| *Gossypium raimondii* | Gorai.008G132000 | Gorai.008G132000.2 | MYB_related | YES |
| *Gossypium raimondii* | Gorai.008G132000 | Gorai.008G132000.3 | MYB_related | YES |
| *Gossypium raimondii* | Gorai.008G132000 | Gorai.008G132000.4 | MYB_related | YES |
| *Gossypium raimondii* | Gorai.008G132000 | Gorai.008G132000.5 | MYB_related | YES |
| *Gossypium raimondii* | Gorai.008G133700 | Gorai.008G133700.1 | MYB | YES |
| *Gossypium raimondii* | Gorai.008G133700 | Gorai.008G133700.2 | MYB | NO |
| *Gossypium raimondii* | Gorai.008G135700 | Gorai.008G135700.1 | SBP | YES |
| *Gossypium raimondii* | Gorai.008G155100 | Gorai.008G155100.1 | HB-other | YES |
| *Gossypium raimondii* | Gorai.008G155100 | Gorai.008G155100.2 | HB-other | YES |
| *Gossypium raimondii* | Gorai.008G155100 | Gorai.008G155100.3 | HB-other | NO |
| *Gossypium raimondii* | Gorai.008G155100 | Gorai.008G155100.4 | HB-other | YES |
| *Gossypium raimondii* | Gorai.008G163200 | Gorai.008G163200.1 | Trihelix | NO |
| *Gossypium raimondii* | Gorai.008G163200 | Gorai.008G163200.2 | Trihelix | NO |
| *Gossypium raimondii* | Gorai.008G163200 | Gorai.008G163200.3 | Trihelix | NO |
| *Gossypium raimondii* | Gorai.008G163200 | Gorai.008G163200.4 | Trihelix | YES |
| *Gossypium raimondii* | Gorai.008G241800 | Gorai.008G241800.1 | GRF | YES |
| *Gossypium raimondii* | Gorai.008G241800 | Gorai.008G241800.2 | GRF | NO |
| *Gossypium raimondii* | Gorai.008G241800 | Gorai.008G241800.3 | GRF | NO |
| *Gossypium raimondii* | Gorai.008G241800 | Gorai.008G241800.4 | GRF | NO |
| *Gossypium raimondii* | Gorai.008G258900 | Gorai.008G258900.1 | HD-ZIP | YES |
| *Gossypium raimondii* | Gorai.008G261400 | Gorai.008G261400.1 | NAC | YES |
| *Gossypium raimondii* | Gorai.008G261400 | Gorai.008G261400.2 | NAC | YES |
| *Gossypium raimondii* | Gorai.008G261400 | Gorai.008G261400.3 | NAC | YES |
| *Gossypium raimondii* | Gorai.008G261400 | Gorai.008G261400.4 | NAC | YES |
| *Gossypium raimondii* | Gorai.008G261400 | Gorai.008G261400.5 | NAC | NO |
| *Gossypium raimondii* | Gorai.008G282200 | Gorai.008G282200.1 | Trihelix | YES |
| *Gossypium raimondii* | Gorai.008G293100 | Gorai.008G293100.1 | MYB | YES |
| *Gossypium raimondii* | Gorai.008G293100 | Gorai.008G293100.2 | MYB | NO |
| *Gossypium raimondii* | Gorai.009G030900 | Gorai.009G030900.1 | MYB_related | NO |
| *Gossypium raimondii* | Gorai.009G030900 | Gorai.009G030900.2 | MYB_related | YES |
| *Gossypium raimondii* | Gorai.009G068400 | Gorai.009G068400.1 | C2H2 | NO |
| *Gossypium raimondii* | Gorai.009G068400 | Gorai.009G068400.2 | C2H2 | NO |
| *Gossypium raimondii* | Gorai.009G068400 | Gorai.009G068400.3 | C2H2 | YES |
| *Gossypium raimondii* | Gorai.009G068400 | Gorai.009G068400.4 | C2H2 | NO |
| *Gossypium raimondii* | Gorai.009G068400 | Gorai.009G068400.5 | C2H2 | NO |
| *Gossypium raimondii* | Gorai.009G070300 | Gorai.009G070300.1 | C3H | YES |
| *Gossypium raimondii* | Gorai.009G070300 | Gorai.009G070300.2 | C3H | NO |
| *Gossypium raimondii* | Gorai.009G083500 | Gorai.009G083500.1 | NAC | YES |
| *Gossypium raimondii* | Gorai.009G092800 | Gorai.009G092800.1 | C2H2 | YES |
| *Gossypium raimondii* | Gorai.009G092800 | Gorai.009G092800.2 | C2H2 | YES |
| *Gossypium raimondii* | Gorai.009G092800 | Gorai.009G092800.3 | C2H2 | YES |
| *Gossypium raimondii* | Gorai.009G097100 | Gorai.009G097100.1 | G2-like | YES |
| *Gossypium raimondii* | Gorai.009G097100 | Gorai.009G097100.2 | G2-like | NO |
| *Gossypium raimondii* | Gorai.009G097100 | Gorai.009G097100.3 | G2-like | NO |
| *Gossypium raimondii* | Gorai.009G097100 | Gorai.009G097100.4 | G2-like | NO |
| *Gossypium raimondii* | Gorai.009G141200 | Gorai.009G141200.1 | C2H2 | YES |
| *Gossypium raimondii* | Gorai.009G141200 | Gorai.009G141200.2 | C2H2 | NO |
| *Gossypium raimondii* | Gorai.009G141200 | Gorai.009G141200.3 | C2H2 | NO |
| *Gossypium raimondii* | Gorai.009G141200 | Gorai.009G141200.4 | C2H2 | YES |
| *Gossypium raimondii* | Gorai.009G141200 | Gorai.009G141200.5 | C2H2 | YES |
| *Gossypium raimondii* | Gorai.009G147300 | Gorai.009G147300.1 | ERF | NO |
| *Gossypium raimondii* | Gorai.009G147300 | Gorai.009G147300.2 | ERF | NO |
| *Gossypium raimondii* | Gorai.009G147300 | Gorai.009G147300.3 | ERF | YES |
| *Gossypium raimondii* | Gorai.009G260000 | Gorai.009G260000.1 | NAC | YES |
| *Gossypium raimondii* | Gorai.009G260000 | Gorai.009G260000.2 | NAC | YES |
| *Gossypium raimondii* | Gorai.009G260000 | Gorai.009G260000.3 | NAC | YES |
| *Gossypium raimondii* | Gorai.009G260000 | Gorai.009G260000.4 | NAC | YES |
| *Gossypium raimondii* | Gorai.009G288000 | Gorai.009G288000.1 | MIKC | YES |
| *Gossypium raimondii* | Gorai.009G288000 | Gorai.009G288000.2 | MIKC | NO |
| *Gossypium raimondii* | Gorai.009G288000 | Gorai.009G288000.3 | MIKC | NO |
| *Gossypium raimondii* | Gorai.009G288000 | Gorai.009G288000.4 | MIKC | NO |
| *Gossypium raimondii* | Gorai.009G288000 | Gorai.009G288000.5 | MIKC | NO |
| *Gossypium raimondii* | Gorai.009G288000 | Gorai.009G288000.6 | MIKC | NO |
| *Gossypium raimondii* | Gorai.009G288000 | Gorai.009G288000.7 | MIKC | NO |
| *Gossypium raimondii* | Gorai.009G288000 | Gorai.009G288000.8 | MIKC | NO |
| *Gossypium raimondii* | Gorai.009G288000 | Gorai.009G288000.9 | MIKC | YES |
| *Gossypium raimondii* | Gorai.009G302800 | Gorai.009G302800.1 | M-type | YES |
| *Gossypium raimondii* | Gorai.009G309000 | Gorai.009G309000.1 | NAC | YES |
| *Gossypium raimondii* | Gorai.009G309200 | Gorai.009G309200.1 | bZIP | YES |
| *Gossypium raimondii* | Gorai.009G309200 | Gorai.009G309200.2 | bZIP | YES |
| *Gossypium raimondii* | Gorai.009G419300 | Gorai.009G419300.1 | S1Fa-like | YES |
| *Gossypium raimondii* | Gorai.009G451700 | Gorai.009G451700.1 | HB-other | YES |
| *Gossypium raimondii* | Gorai.009G451700 | Gorai.009G451700.2 | HB-other | NO |
| *Gossypium raimondii* | Gorai.009G451700 | Gorai.009G451700.3 | HB-other | YES |
| *Gossypium raimondii* | Gorai.009G451700 | Gorai.009G451700.4 | HB-other | YES |
| *Gossypium raimondii* | Gorai.009G451700 | Gorai.009G451700.5 | HB-other | NO |
| *Gossypium raimondii* | Gorai.009G451700 | Gorai.009G451700.7 | HB-other | NO |
| *Gossypium raimondii* | Gorai.010G025100 | Gorai.010G025100.1 | LBD | NO |
| *Gossypium raimondii* | Gorai.010G025100 | Gorai.010G025100.2 | LBD | YES |
| *Gossypium raimondii* | Gorai.010G132400 | Gorai.010G132400.1 | M-type | YES |
| *Gossypium raimondii* | Gorai.010G197800 | Gorai.010G197800.1 | NF-X1 | YES |
| *Gossypium raimondii* | Gorai.010G197800 | Gorai.010G197800.2 | NF-X1 | YES |
| *Gossypium raimondii* | Gorai.010G222900 | Gorai.010G222900.1 | ERF | YES |
| *Gossypium raimondii* | Gorai.010G243500 | Gorai.010G243500.1 | HB-other | YES |
| *Gossypium raimondii* | Gorai.010G243500 | Gorai.010G243500.2 | HB-other | YES |
| *Gossypium raimondii* | Gorai.010G243500 | Gorai.010G243500.3 | HB-other | YES |
| *Gossypium raimondii* | Gorai.010G243500 | Gorai.010G243500.4 | HB-other | YES |
| *Gossypium raimondii* | Gorai.010G247100 | Gorai.010G247100.1 | G2-like | YES |
| *Gossypium raimondii* | Gorai.010G247100 | Gorai.010G247100.2 | G2-like | NO |
| *Gossypium raimondii* | Gorai.010G247100 | Gorai.010G247100.3 | G2-like | NO |
| *Gossypium raimondii* | Gorai.010G247100 | Gorai.010G247100.4 | G2-like | NO |
| *Gossypium raimondii* | Gorai.010G247100 | Gorai.010G247100.5 | G2-like | NO |
| *Gossypium raimondii* | Gorai.010G247100 | Gorai.010G247100.6 | G2-like | NO |
| *Gossypium raimondii* | Gorai.010G247100 | Gorai.010G247100.7 | G2-like | NO |
| *Gossypium raimondii* | Gorai.010G256900 | Gorai.010G256900.1 | HB-other | YES |
| *Gossypium raimondii* | Gorai.010G256900 | Gorai.010G256900.2 | HB-other | YES |
| *Gossypium raimondii* | Gorai.010G256900 | Gorai.010G256900.3 | HB-other | YES |
| *Gossypium raimondii* | Gorai.010G256900 | Gorai.010G256900.4 | HB-other | YES |
| *Gossypium raimondii* | Gorai.010G256900 | Gorai.010G256900.5 | HB-other | YES |
| *Gossypium raimondii* | Gorai.010G256900 | Gorai.010G256900.6 | HB-other | YES |
| *Gossypium raimondii* | Gorai.011G029400 | Gorai.011G029400.1 | SBP | YES |
| *Gossypium raimondii* | Gorai.011G029400 | Gorai.011G029400.2 | SBP | NO |
| *Gossypium raimondii* | Gorai.011G035500 | Gorai.011G035500.1 | MIKC | YES |
| *Gossypium raimondii* | Gorai.011G035500 | Gorai.011G035500.2 | MIKC | NO |
| *Gossypium raimondii* | Gorai.011G035500 | Gorai.011G035500.3 | MIKC | NO |
| *Gossypium raimondii* | Gorai.011G035500 | Gorai.011G035500.4 | MIKC | NO |
| *Gossypium raimondii* | Gorai.011G035500 | Gorai.011G035500.5 | MIKC | YES |
| *Gossypium raimondii* | Gorai.011G035500 | Gorai.011G035500.6 | MIKC | NO |
| *Gossypium raimondii* | Gorai.011G035500 | Gorai.011G035500.7 | MIKC | NO |
| *Gossypium raimondii* | Gorai.011G035500 | Gorai.011G035500.8 | MIKC | NO |
| *Gossypium raimondii* | Gorai.011G035500 | Gorai.011G035500.9 | MIKC | NO |
| *Gossypium raimondii* | Gorai.011G069200 | Gorai.011G069200.1 | bHLH | YES |
| *Gossypium raimondii* | Gorai.011G150200 | Gorai.011G150200.1 | bHLH | NO |
| *Gossypium raimondii* | Gorai.011G150200 | Gorai.011G150200.2 | bHLH | NO |
| *Gossypium raimondii* | Gorai.011G150200 | Gorai.011G150200.3 | bHLH | YES |
| *Gossypium raimondii* | Gorai.011G158700 | Gorai.011G158700.1 | M-type | YES |
| *Gossypium raimondii* | Gorai.011G170200 | Gorai.011G170200.1 | G2-like | YES |
| *Gossypium raimondii* | Gorai.011G183800 | Gorai.011G183800.1 | SBP | YES |
| *Gossypium raimondii* | Gorai.011G183800 | Gorai.011G183800.2 | SBP | NO |
| *Gossypium raimondii* | Gorai.011G195800 | Gorai.011G195800.1 | FAR1 | YES |
| *Gossypium raimondii* | Gorai.011G195800 | Gorai.011G195800.2 | FAR1 | YES |
| *Gossypium raimondii* | Gorai.011G195800 | Gorai.011G195800.3 | FAR1 | YES |
| *Gossypium raimondii* | Gorai.011G195800 | Gorai.011G195800.4 | FAR1 | YES |
| *Gossypium raimondii* | Gorai.011G195800 | Gorai.011G195800.5 | FAR1 | YES |
| *Gossypium raimondii* | Gorai.011G195800 | Gorai.011G195800.6 | FAR1 | YES |
| *Gossypium raimondii* | Gorai.011G198600 | Gorai.011G198600.1 | CAMTA | YES |
| *Gossypium raimondii* | Gorai.011G198600 | Gorai.011G198600.2 | CAMTA | YES |
| *Gossypium raimondii* | Gorai.011G198600 | Gorai.011G198600.3 | CAMTA | YES |
| *Gossypium raimondii* | Gorai.011G198600 | Gorai.011G198600.4 | CAMTA | YES |
| *Gossypium raimondii* | Gorai.011G198600 | Gorai.011G198600.5 | CAMTA | YES |
| *Gossypium raimondii* | Gorai.011G198600 | Gorai.011G198600.6 | CAMTA | YES |
| *Gossypium raimondii* | Gorai.011G198600 | Gorai.011G198600.7 | CAMTA | YES |
| *Gossypium raimondii* | Gorai.011G204700 | Gorai.011G204700.1 | CAMTA | NO |
| *Gossypium raimondii* | Gorai.011G204700 | Gorai.011G204700.2 | CAMTA | NO |
| *Gossypium raimondii* | Gorai.011G204700 | Gorai.011G204700.3 | CAMTA | NO |
| *Gossypium raimondii* | Gorai.011G204700 | Gorai.011G204700.4 | CAMTA | NO |
| *Gossypium raimondii* | Gorai.011G204700 | Gorai.011G204700.5 | CAMTA | NO |
| *Gossypium raimondii* | Gorai.011G204700 | Gorai.011G204700.6 | CAMTA | YES |
| *Gossypium raimondii* | Gorai.012G007100 | Gorai.012G007100.1 | bZIP | YES |
| *Gossypium raimondii* | Gorai.012G007100 | Gorai.012G007100.2 | bZIP | NO |
| *Gossypium raimondii* | Gorai.012G007100 | Gorai.012G007100.3 | bZIP | NO |
| *Gossypium raimondii* | Gorai.012G009900 | Gorai.012G009900.1 | C3H | NO |
| *Gossypium raimondii* | Gorai.012G009900 | Gorai.012G009900.2 | C3H | NO |
| *Gossypium raimondii* | Gorai.012G009900 | Gorai.012G009900.3 | C3H | NO |
| *Gossypium raimondii* | Gorai.012G009900 | Gorai.012G009900.4 | C3H | NO |
| *Gossypium raimondii* | Gorai.012G009900 | Gorai.012G009900.5 | C3H | NO |
| *Gossypium raimondii* | Gorai.012G009900 | Gorai.012G009900.6 | C3H | NO |
| *Gossypium raimondii* | Gorai.012G009900 | Gorai.012G009900.7 | C3H | YES |
| *Gossypium raimondii* | Gorai.012G055400 | Gorai.012G055400.1 | HD-ZIP | YES |
| *Gossypium raimondii* | Gorai.012G055400 | Gorai.012G055400.2 | HD-ZIP | YES |
| *Gossypium raimondii* | Gorai.012G125500 | Gorai.012G125500.1 | NAC | NO |
| *Gossypium raimondii* | Gorai.012G125500 | Gorai.012G125500.2 | NAC | YES |
| *Gossypium raimondii* | Gorai.012G176500 | Gorai.012G176500.1 | SBP | NO |
| *Gossypium raimondii* | Gorai.012G176500 | Gorai.012G176500.2 | SBP | NO |
| *Gossypium raimondii* | Gorai.012G176500 | Gorai.012G176500.3 | SBP | NO |
| *Gossypium raimondii* | Gorai.012G176500 | Gorai.012G176500.4 | SBP | YES |
| *Gossypium raimondii* | Gorai.012G180300 | Gorai.012G180300.1 | MIKC | YES |
| *Gossypium raimondii* | Gorai.012G180300 | Gorai.012G180300.2 | MIKC | YES |
| *Gossypium raimondii* | Gorai.013G000800 | Gorai.013G000800.1 | bZIP | YES |
| *Gossypium raimondii* | Gorai.013G035200 | Gorai.013G035200.1 | bZIP | YES |
| *Gossypium raimondii* | Gorai.013G061100 | Gorai.013G061100.1 | CAMTA | NO |
| *Gossypium raimondii* | Gorai.013G061100 | Gorai.013G061100.2 | CAMTA | NO |
| *Gossypium raimondii* | Gorai.013G061100 | Gorai.013G061100.3 | CAMTA | YES |
| *Gossypium raimondii* | Gorai.013G080200 | Gorai.013G080200.1 | G2-like | YES |
| *Gossypium raimondii* | Gorai.013G139200 | Gorai.013G139200.1 | MYB_related | NO |
| *Gossypium raimondii* | Gorai.013G139200 | Gorai.013G139200.2 | MYB_related | YES |
| *Gossypium raimondii* | Gorai.013G179400 | Gorai.013G179400.1 | WRKY | NO |
| *Gossypium raimondii* | Gorai.013G179400 | Gorai.013G179400.2 | WRKY | YES |
| *Gossypium raimondii* | Gorai.013G179400 | Gorai.013G179400.3 | WRKY | NO |
| *Gossypium raimondii* | Gorai.013G179400 | Gorai.013G179400.4 | WRKY | NO |
| *Gossypium raimondii* | Gorai.013G204300 | Gorai.013G204300.1 | bHLH | YES |
| *Gossypium raimondii* | Gorai.N017200 | Gorai.N017200.1 | MIKC | YES |
| *Gossypium raimondii* | Gorai.N017200 | Gorai.N017200.2 | MIKC | NO |
| *Gossypium raimondii* | Gorai.N017200 | Gorai.N017200.3 | MIKC | NO |
